# Emotional Labor in Health Care: The Moderating Roles of Personality and the Mediating Role of Sleep on Job Performance and Satisfaction

**DOI:** 10.3389/fpsyg.2020.574898

**Published:** 2020-12-17

**Authors:** Shu-Chuan Jennifer Yeh, Shih-Hua Sarah Chen, Kuo-Shu Yuan, Willy Chou, Thomas T. H. Wan

**Affiliations:** ^1^Department of Business Management, National Sun Yat-sen University, Kaohsiung, Taiwan; ^2^Division of Social Science, University of Chicago, Chicago, IL, United States; ^3^School of Business Administration, Huaqiao University, Quanzhou, China; ^4^Department of Physical Medicine and Rehabilitation, Chi Mei Medical Center, Tainan, Taiwan; ^5^Department of Physical Medicine and Rehabilitation, Chong Shan University, Taichung, Taiwan; ^6^Department of Health Management and Informatics, University of Central Florida, Orlando, FL, United States

**Keywords:** emotional labor, sleep problems, personality traits, job satisfaction, job performance

## Abstract

The objective of this study is to investigate the effects of emotional labor on job performance and satisfaction, as well as to examine the mediating effect of sleep problems and the moderating effects of personality traits. A time-lagged study was conducted on 864 health professionals. Scales for emotional labor, sleep, personality traits, and job satisfaction were used and job performance data was obtained from records maintained by human resources. Structural equation modeling was performed to investigate the relations. Sleep problems only partially mediated the relationship between surface acting and job satisfaction but completely mediated the relationship between surface acting and job performance. Several personality traits were shown to moderate the relationship between surface acting and sleep problems. The effects were stronger for people with low agreeableness and high neuroticism. The relationship between high levels of deep acting and low levels of sleep problems was more pronounced in individuals with low extraversion. Supervisors should be conscious of emotional labor in the work context and provide necessary deep acting training to facilitate emotional regulation.

## Introduction

Emotional labor is a means for employees to manage their emotions and to express only those requested by their organizations (Hochschild, 1983). Notably, in a service-oriented sector, employers often ask employees to display certain favorable emotions. To exhibit the required emotional responses, employees generally vary on two dimensions of emotional labor – surface acting or deep acting – to regulate their emotions. Surface acting is a person’s untruthful appearance of a prescribed emotion without attempting to change their authentic feelings, eventually leading to emotional exhaustion (Martínez-Iñigo et al., 2007). Deep acting refers to demonstrating a person’s authentic emotions (Ashforth and Humphrey, 1993; Grandey, 2003; Groth et al., 2009), and is often found unrelated to employee stress or physical well-being (Grandey, 2003).

Working with people is likely to involve a significant amount of emotional labor. The characteristics and context of healthcare render emotional labor inevitable for healthcare professionals. Healthcare workers use emotional labor to create and cultivate bonds with patients or clients (Fouquereau et al., 2019), in particular when they wish to reduce patients’ anxiety or need to perform an unpleasant procedure (Martínez-Iñigo et al., 2007). Gray (2010) advocates that emotional labor is a core component of nurses’ role in making patients feel safe and comfortable. The surge of research on emotional labor has demonstrated its pervasive effects on organizational outcomes (Grandey and Sayre, 2019). Previous studies have demonstrated the direct effects of emotional labor (or emotional dissonance) on organizational outcomes (Kammeyer-Mueller et al., 2013; Deng et al., 2017; Grandey and Sayre, 2019) and employee well-being (Hintsanen et al., 2014; Wagner et al., 2014; Diestel et al., 2015; Grandey and Sayre, 2019). However, relatively few studies have investigated the mechanism between emotional labor and work outcomes (Wagner et al., 2014; Deng et al., 2017). Notably, Deng et al. (2017) explored the mechanism of ego depletion between emotional labor and coworker harmful behavior, whereas Wagner et al. (2014) examined the mechanism of anxiety between surface acting and emotional exhaustion and insomnia.

The present study focuses on the impact of emotional labor on sleep, and how sleep problems can further influence job satisfaction and job performance. In particular, we argue that emotional labor is a predictive factor of sleep, which indirectly influences work outcomes. The current literature also proposes that personality may be related to sleep (Hintsanen et al., 2014; Kim et al., 2015; Allen et al., 2016) and emotional labor (Yagil and Medler-Liraz, 2017). Drawing on these studies, this research integrates the theories of emotional labor, sleep, and personality. We posit the moderating role of personality traits and examine how they adjust the relationship between emotional labor and sleep. In doing so, we highlight the need for a more comprehensive investigation of the topic (Kammeyer-Mueller et al., 2013; Grandey and Gabriel, 2015).

Accordingly, this study aims to extend the theoretical and empirical approaches to emotional labor by delineating the degree and mediating effect of sleep problems on the relationship between emotional labor and job satisfaction and performance. We propose that some people replenish their psychological resources more quickly than others, which can be attributed to variances in personality traits. Thus, we theorize that employee personality traits (i.e., conscientiousness, neuroticism, agreeableness, extraversion, and openness) moderate the relationship between emotional labor and sleep problems. Identifying these moderators will help offer insights on the processes of emotional labor.

## Literature Review and Hypotheses

Many service-oriented jobs require specific displayed affects from employees, that is, the expression of positive affect (e.g., cheerfulness) and the suppression of negative affect (e.g., annoyance) (Groth et al., 2009). Negative effects have been negatively associated with falling asleep, maintaining sleep, and reinitiating sleep (Fortunato and Harsh, 2006). When faced with affective requirements, the emotional regulation strategies adopted by employees can impact their sleep patterns. For instance, Wagner et al. (2014) found surface acting conducted at work to be associated with insomnia experienced during nighttime.

On the other hand, employees experiencing emotional requirements can decide to fulfill the displayed rules by making efforts to align real and required emotions. However, excessive efforts to regulate one’s emotions can lead to ego depletion (Baumeister et al., 1998) and feelings of exhaustion, which negatively impacts sleep quality (Baglioni et al., 2010). Incongruence between felt and required emotions when surface acting creates tension and emotional dissonance (Pugh et al., 2011). Individuals who regularly endure stress associated with surface acting are more prone to depression and anxiety, which decrease job performance and contribute to burnout (Weaver et al., 2019). Such dissonance (i.e., surface acting) results in sleep problems, making it increasingly difficult to control emotions and thus, triggering a vicious cycle between emotional labor and poor sleep (Palmer and Alfano, 2017).

Sleep quality is crucial for optimal brain function (Saper et al., 2005). High sleep quality stabilizes the cerebral metabolic rate and ensures adequate resources for the prefrontal cortex (Hofmann et al., 2012). In contrast, sleep loss is likely to affect psychological well-being (Diestel et al., 2015) and organizational outcomes, including safety (Weaver et al., 2019), job satisfaction (Scott and Judge, 2006), and performance (Amirian, 2014). Exploring the relationships among insomnia, emotions, and job satisfaction for 45 employees, Scott and Judge (2006) discovered that insomnia is negatively correlated with job satisfaction. Tomasko et al. (2012), however, demonstrated no difference in the performance or learning of surgical tasks between sleep-deprived medical students and the control group, although the former reported an increase in total subjective mental workload. In a study of 171 nurses and 75 junior and senior business students, Christian and Ellis (2011) found that sleep problems reduce self-control and increase hostility, resulting in heightened workplace deviance.

Deep acting produces a natural and genuine change in emotions, and the individual engaging in it may be considered more trustworthy by customers, clients, and patients (Groth et al., 2009). Unlike surface acting, deep acting does not consume as many psychological resources (Goldberg and Grandey, 2007), given the lower levels of incongruence between felt and displayed emotions (Cheung et al., 2018; Fouquereau et al., 2019). Past evidence suggested that stronger deep acting is associated with lower cognitive exhaustion (Xanthopoulou et al., 2018). Lower cognitive exhaustion is related to better sleep (Hülsheger and Shewe, 2011) and a reduced need for recovery at the end of workdays (Xanthopoulou et al., 2018). Thus, we can conclude that deep acting is negatively associated with sleep problems. Integrating the findings of these studies, we hypothesize the following:

Hypothesis 1a: Sleep problems mediate the relationship between surface acting and job satisfaction (job performance), wherein surface acting is positively associated with sleep problems, which in turn, are negatively associated with job satisfaction (job performance).

Hypothesis 1b: Sleep problems mediate the relationship between deep acting and job satisfaction (job performance), wherein deep acting is negatively associated with sleep problems, which in turn, are positively associated with job satisfaction (job performance).

Individuals show various responses and employ different strategies to cope with stress, and thus, we expect work outcomes to vary by individual personality. Individuals who regulate their emotions through deep acting may remain connected to their core values and beliefs, resulting in an alignment of their felt and shown emotions. By contrast, individuals who resort to surface acting may show incongruence between felt and shown emotions. This study delineates the way emotional labor strategies combine with specific employees’ personality traits to influence their sleep quality and, in turn, job outcomes to understand the mechanism between emotional labor, sleep, and work outcomes.

Personality factors have been linked to the development of sleep problems. People with high conscientiousness live longer because they participate in more healthy activities, which include regular exercise, healthy diets, avoidance of substance abuse, and fewer risk behaviors (Bogg and Roberts, 2004). Using different measurement scales, several studies have found that higher conscientiousness is associated with improved daily sleep quality and sleep efficiency (O’Connor, 2014; Kim et al., 2015). Highly conscientious individuals practice healthy lifestyles that help regulate their sleep quality. Therefore, when individuals high in conscientiousness engage in surface acting, their conscientiousness traits have a protective effect between surface acting and sleep problems. Thus, we hypothesize the following:

Hypothesis 2: Conscientiousness moderates the positive relationship between surface acting and sleep problems, and the effect is weaker for individuals with high conscientiousness.

Individuals with neuroticism are characterized as more anxious and stressed during interactions, and thus, are less likely to experience positive emotions (Kiffin-Petersen et al., 2011). Such individuals may need to alter their feelings to maintain positive displayed emotions by engaging in more surface acting or deep acting. Studies have shown that neurotic individuals engage in more surface acting (Diefendorff et al., 2005; Kiffin-Petersen et al., 2011). Most individuals with high neuroticism tend to exhibit fewer health-promoting behaviors (Booth-Kewley and Vickers, 1994) and are more susceptible to and more likely to report somatic complaints (Watson and Clark, 1994) possibly related to poor sleep quality (Williams and Moroz, 2009; Cellini et al., 2017). Performing affective requirements and disguising real emotions will harm individuals’ sleep activities. This could exacerbate sleep problems among individuals with high neuroticism who are already more susceptible to negative emotions than individuals with low neuroticism. Therefore, we hypothesize the following:

Hypothesis 3: Neuroticism moderates the positive relationship between surface acting and sleep problems, and this effect is stronger for individuals with high neuroticism.

Extraversion is characterized by warmth, cheerfulness, or vigor and is used to express enthusiasm or interest. Extraverts are more open to social influences, suggesting that they may be more willing to engage in the emotions required by their organizations (Wilson, 1981), and experience more positive emotions (Watson and Clark, 1994; Judge et al., 2009). Bono and Vey (2007) found that individuals who score high on extraversion (rather than low) respond better to organizational demands for positive emotions through deep acting. High extraverts can easily engage in deep acting as they naturally have mood traits that align with the required emotions (Diefendorff et al., 2005). Judge et al. (2009) further demonstrated that deep acting generates more positive reactions for extraverts.

Extraversion is associated with shorter sleep latency (Williams and Moroz, 2009) and better sleep quality (Hintsanen et al., 2014; Allen et al., 2016), but not with sleep duration (Williams and Moroz, 2009). By provoking desired feelings (Chi et al., 2011) and a good-faith attempt to produce internal emotions, deep acting shows less of an influence on sleep. In combination, high extraversion and deep acting engender more positive affect, thereby promoting the expression of more naturally felt emotions – and in turn, reducing sleep problems. We therefore hypothesize that extraversion moderates the negative relationship between deep acting and sleep problems.

Hypothesis 4: Extraversion moderates the negative relationship between deep acting and sleep problems, and this effect is stronger for people with high extraversion.

Agreeableness is the tendency to be compassionate, kind, and considerate toward others (John and Srivastava, 1999). Agreeableness is congruent with the positive requirement of service jobs and is positively related with deep acting (Diefendorff and Richard, 2003; Kammeyer-Mueller et al., 2013). Findings on the relationship between agreeableness and sleep problems are mixed. Several studies find no relation (Williams and Moroz, 2009; Stephan et al., 2018), while others show that lower agreeableness is related to fewer sleeping hours (Randler, 2008) and higher sleep deficiency (Hintsanen et al., 2014; Cellini et al., 2017). Employing surface acting to regulate emotions increases sleep problems. The interplay of surface acting and low agreeableness exacerbates the situation (Törnroos et al., 2012). Thus, we hypothesize the following:

Hypothesis 5: Agreeableness moderates the positive relationship between surface acting and sleep problems, and this effect is stronger for people with low agreeableness.

Individuals with openness can be described as imaginative, curious and open-minded toward new experiences. Studies have been inconclusive about the relationship among openness, emotional labor, and sleep. Though several studies report that openness is not associated with sleep quality (Hintsanen et al., 2014; Cellini et al., 2017; Stephan et al., 2018), Allen et al. (2016) found that higher openness is related to sleep difficulties. The abovementioned studies, however, do not examine the moderating effect of openness. Openness is generally considered to be an intrapsychic trait rather than an interpersonal trait (Costa and McCrae, 2012). Individuals high on openness to experience generally practice more deep acting than surface acting and, thus, are less emotionally exhausted in stressful situations (Zapf, 2002) and do not burn out easily (Bakker et al., 2002; Divinakumar et al., 2019). We, therefore, hypothesize the following:

Hypothesis 6: Openness to experiences moderates the negative relationship between deep acting and sleep problems, and this effect is stronger for people with high openness to experiences.

**FIGURE 1:**
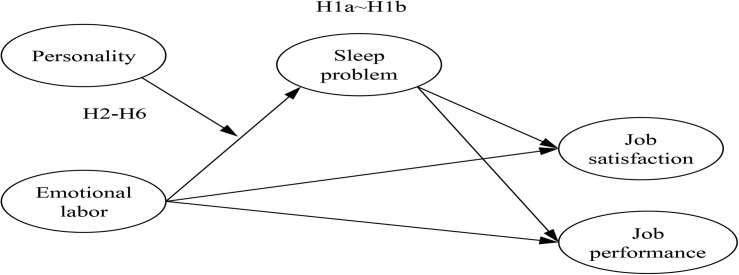
Conceptual model of this study.

Figure 1 summarizes our hypotheses.

## Materials and Methods

### Procedures and Participants

This study utilized a time-lagged survey. The questionnaire included subscales for emotional labor, sleep issues, personality, and job satisfaction. Questionnaires with an accompanying letter describing the study were distributed to full-time healthcare professionals who provide direct care to patients (*n* = 1,061), including 321 physicians, 530 nurses, and 210 allied health professionals. To reduce common methods variance, the surveys were directly returned to the principal investigator in sealed envelopes at three time periods in 2016. The measures for emotional labor, sleep problems, and personality traits and job satisfaction were returned in March, May, and July 2016, respectively. This study was approved by the institutional review board of the study hospital. Written informed consent was obtained from all participants prior to the first data collection.

A total of 990 questionnaires were returned, of which 126 were incomplete. The final sample of 864 valid questionnaires (81.43% response rate) was composed of 206 physicians, 495 nurses, and 163 allied healthcare professionals. In addition, we collected data on objective job performance for 2016 from the human resources department. The mean age of participants was 34.25 years. About 70.9% were female and 54.5% were single. The average tenure was 8.64 years. Finally, 84.6% had college degrees and 12.6% were supervisors.

### Measures

#### Job Satisfaction

Job satisfaction was measured on the basis of six items adopted from Brayfield and Rothe (1951). The respondents rated their satisfaction on a five-point Likert scale, where 1 was “strongly disagree” and 5 was “strongly agree.” An example item was “I like my job better than the average worker does.” Responses to these items were averaged. The alpha reliability in this study was α = .91.

#### Job Performance

*Job performance* scores were obtained from human resource records on the respondents’ performance appraisals provided by their supervisors. These scores include five categories: 1 (below 0.5), 2 (0.75), 3 (1.0), 4 (1.25), and 5 (1.5 and above), with higher scores indicating better performance. Performance bonus base are shown in the parentheses. Since the objective performance scores were rated by unit supervisors, we examined intraclass correlations (ICCs) (LaHuis and Avis, 2007). Each employee’s job performance was rated by one supervisor within their department. ICC(1) was used to examine if there exists between-department differences – as performance evaluations may be more similar within the same department having been rated by the same supervisors and differ more across different departments. An ICC (1) of 0.06, as demonstrated in this study, evidences that the effect of between-department difference is small, and job performance is likely not influenced by higher-level factors.

#### Emotional Labor

The measure for *emotional labor* asked employees to measure the level of deep and surface acting using the scales developed by Brotheridge and Lee (2002). It contained three items to measure surface acting (e.g., “Pretend to have emotions that you do not really have”; α = 0.72) and three items to measure deep acting (e.g., “Make an effort to actually feel the emotions that you need to display to others”; α = 0.85). The items were scored on a five-point Likert scale, where 1 was “strongly disagree” and 5 was “strongly agree.” The alpha reliability was 0.72 and 0.85 for surface acting and deep acting, respectively.

#### Sleep Problems

Sleep problems were assessed using the Pittsburgh Sleep Quality Index (PSQI) (Buysse et al., 1989). The PSQI is a four-point Likert scale survey containing 21 items that each fall into one of the following seven components: subjective sleep quality, sleep latency, sleep duration, habitual sleep efficiency, sleep disturbances, use of sleeping medication, and daytime dysfunction. A score (ranging from 0 – no difficulty to 3 – severe difficulty) is derived for each of the seven components prior to summing the component scores to produce a final global PSQI score (range 0–21) (Buysse et al., 1989). Higher scores indicate lower sleep quality. The PSQI has been shown to have good reliability, with a coefficient value of 0.81 for the present study.

#### Personality

Personality traits were assessed using the big five inventory (BFI) personality questionnaire (John and Srivastava, 1999). The scale contains 44 items with five constructs: extraversion (8 items; e.g., “I see myself as someone who is full of energy”), neuroticism (8 items; e.g., “I see myself as someone who is relaxed and handles stress well”), agreeableness (9 items; e.g., “I see myself as someone who is considerate and kind to almost everyone”), conscientiousness (9 items; e.g., “I see myself as someone who is a reliable worker”), and openness (10 items; e.g., “I see myself as someone who likes to reflect and play with ideas”). Each item was rated using a five-point Likert scale, where 1 was “strongly disagree” and 5 was “strongly agree.” In the present study, the internal consistencies for extraversion, agreeableness, conscientiousness, neuroticism, and openness were 0.80, 0.79, 0.82, 0.82, and 0.75, respectively.

#### Control Variables

Other measures associated with job satisfaction and job performance were considered as control variables. Previous research indicated that higher levels of formal education are associated with better job performance (Ng and Feldman, 2009) and job satisfaction (Fabra and César Camisón, 2009). Hence, we controlled for education [coded as “vocational school” (1), “college” (2), and “graduate’ (3)]. We created a tenure variable (in years) to control for job experience, assuming that years on the job is predictive of job performance (Schmidt et al., 1986). Research has also demonstrated that older workers have few motives to join training programs as compared to younger workers; hence, we controlled for age (in years) due to its’ influence on performance (Maurer et al., 2003).

The relationship between marital status and job performance is complex. Married men earn 12.4 percent (Chun and Lee, 2001) more and attain better performance than single men (Mehay and Bowman, 2005). Thus, we controlled for marital status (coded as “0 = single” and “1 = married”). Health care professionals with managerial positions are usually responsible for supervising other employees and can influence unit performance. Supervisory duties include hiring and firing, performing employee evaluations, and monitoring employee work performance. Thus, we controlled for managerial position (coded as “1 = yes” and “0 = no”). Job performance and job satisfaction are quite different across professional groups, while predicting factors for job satisfaction and performance are also diverse. For example, professional development, supervisor support, and local leadership have been shown to be main predictors of job satisfaction for physicians, nurses, and auxiliaries (Krogstad et al., 2006). Hence, we also controlled for professional type (1 = nurses, 2 = allied health professionals, and 3 = physicians). We did not control for race since a majority of the participants are of the same race.

### Analyses

We used structural equation modeling (SEM) in AMOS 18.0 (SPSS Inc, 2009) to test the hypotheses. After establishing an appropriate measurement model, the structural models for the direct, indirect, and moderating effects were estimated. We controlled for the effects of age, gender, marital status, education, professional type, position, and tenure in all the structural models by adding direct paths to three endogenous variables (sleep problems, job satisfaction, and job performance) while allowing the models to co-vary with all other exogenous latent variables. To assess the significance of the mediating effects, we also examined the significance and the bias-corrected confidence intervals of the direct, indirect, and total effects generated from 5,000 samples using bootstrapping procedures (Klein and Moosbrugger, 2000).

Next, we formed product terms for use in SEM to provide estimates for the moderating effects of latent constructs for each of the five personality traits. The parceling procedure was adopted in this study as this technique is commonly used and increases the overall stability of a model (Landis et al., 2000; Little et al., 2002). Parceling creates a smaller set of indicators, formed by randomly grouping items within each scale to serve as indicators of the latent variables (Bentler and Chou, 1987; Williams and Anderson, 1994). We created three parcels for each of the latent constructs which have more than three items, including five personality traits (extraversion, agreeableness, conscientiousness, neuroticism, and openness).

We followed Jaccard and Wan’s (1995) mean-centering procedure to multiply the main effect indicators of emotional labor (surface acting and deep acting) and personality traits (extraversion, agreeableness, conscientiousness, neuroticism, and openness to experience) to reduce the multicollinearity between the main effects and product terms. In addition, we used multiple product term indicators to obtain the latent interaction terms. We analyzed the moderating effect of personality traits by identifying the significance of the effect of the product terms for emotional labor and personality traits on job satisfaction and job performance.

We used the following model fit indices to evaluate the adequacy of the measurement and structural models: chi-square goodness-of-fit statistic, goodness-of-fit index (GFI), comparative fit index (CFI), Tucker–Lewis index (TLI), root-mean-square error of approximation (RMSEA), and standardized root-mean-square residual (SRMR) (Bentler, 1995; Tucker and Lewis, 1973). The chi-square difference test was used to compare the fit of each hypothesized structural model with that of the alternative model (Jöreskog and Sörbom, 1993).

## Results

Table 1 presents the means, standard deviations, and bivariate correlations for this study’s variables. First, a confirmatory factor analysis is performed to validate each measurement model. Table 2 shows the baseline measurement model (Model 1) and that all study variables loaded on their respective factors, except sleep problems (observed variable), have a good fit [χ^2^ (d.f. = 224) = 863.164, GFI = 0.925, CFI = 0.926, TLI = 0.909, RMSEA = 0.056, and SRMR = 0.052]. Second, the one-factor model (Model 2) wherein all the variables are loaded on one global factor report poor fit [χ^2^ (d.f. = 252) = 5371.357, GFI = 0.641, CFI = 0.384, TLI = 0.325, RMSEA = 0.153, and SRMR = 0.134]. Finally, to evaluate the discriminant validity of the latent constructs, we compare the baseline model with the one-factor model. The baseline model has a significantly lower χ^2^ value, yielding an excellent fit [Δχ^2^ (d.f. = 28) = 4508.19, *p* < 0.001]. This evidences the discriminant validity of our measures for the latent constructs. We also computed average variance extracted (AVE) with the squared correlation and find the square root of the AVE for each latent variable exceeds the correlations between the constructs. The discriminant validities are then established (Table 1).

**TABLE 1:** Means, standard deviations, and correlations^a,b^.

Variables	M	s.d.	1	2	3	4	5	6	7	8	9	10	11	12
01. Age	34.25	7.78	–											
02. Tenure	8.64	6.53	0.77***	–										
03. Surface Acting	7.64	1.97	−0.14**	−0.11**	(0.72)	[0.70]								
04. Deep Acting	9.72	2.18	−0.02	−0.01	0.15**	(0.85)	[0.82]							
05. Extraversion	26.25	4.34	0.01	0.02	−0.13**	0.12**	(0.80)	[0.76]						
06. Agreeableness	32.83	4.40	0.13**	0.16**	−0.20**	0.10**	0.20**	(0.79)	[0.77]					
07. Conscientiousness	30.57	4.87	0.25**	0.25**	−0.19**	0.13**	0.26**	0.37**	(0.82)	[0.71]				
08. Neuroticism	22.88	4.77	−0.18**	−0.13**	0.19**	−0.04	−0.35**	−0.33**	−0.41**	(0.82)	[0.70]			
09. Openness	32.44	4.42	0.12**	0.04	0.01	0.14**	0.33**	0.24**	0.31**	−0.21**	(0.75)	[0.70]		
10. Sleep problem	6.14	2.92	−0.09**	0.03	0.09*	−0.002	−0.05	−0.002	−0.05	0.27**	−0.13**	(0.81)	[-]	
11. Job Satisfaction	24.12	5.36	0.21**	0.19**	−0.14**	0.12**	0.28**	0.28**	0.23**	−0.26**	0.17**	−0.13**	(.91)	[.87]
12. Job Performance	2.86	0.67	0.14*	0.05	−0.05	0.02	0.07**	0.06	0.11**	−0.08*	0.05	−0.13**	.05	-

^*a*^Coefficient alphas appear on the diagonal in parentheses. ^*b*^The square root of average variance extracted (AVE) are in square brackets on the diagonal. *p < 0.05; **p < 0.01.

**TABLE 2:** Measurement and structural model tests.

Models	χ^2^	Δχ^2^	*df*	GFI	CFI	TLI	RMSEA	SRMR
**Measurement Models**								
Model 1: Baseline measurement model	863.164		224	0.925	0.926	0.909	0.056	0.052
Model 2: One-factor model	5371.357	4508.193***	252	0.641	0.384	0.325	0.153	0.134
Structural Models								
Model 3: Baseline structural model	216.421		91	0.976	0.983	0.964	0.040	0.025
Model 4: Latent interaction model – the moderating effect of E	473.830	257.409***	259	0.965	0.980	0.968	0.031	0.028
Model 5: Latent interaction model – the moderating effect of A	476.717	260.296***	259	0.964	0.977	0.964	0.031	0.034
Model 6: Latent interaction model – the moderating effect of C	535.786	319.365***	259	0.959	0.972	0.957	0.035	0.035
Model 7: Latent interaction model – the moderating effect of N	582.223	365.802***	259	0.956	0.969	0.951	0.038	0.037
Model 8: Latent interaction model – the moderating effect of O	449.796	449.796**	259	0.966	0.981	0.971	0.029	0.028

*df, degrees of freedom; GFI, Goodness-of-fit index; CFI, Comparative fit Index; TLI, Tucker–Lewis index; RMSEA, root mean square error of approximation; SRMR, Standardized root mean square residual; E, Extraversion; C, Conscientiousness; A, Agreeableness; N, Neuroticism; O, Openness. Baseline measurement model (model 1) vs on-factor model (model 2), Δχ^2^(28) = 4508.193, *p* < 0.001.*

### Mediating Effects

**FIGURE 2:**
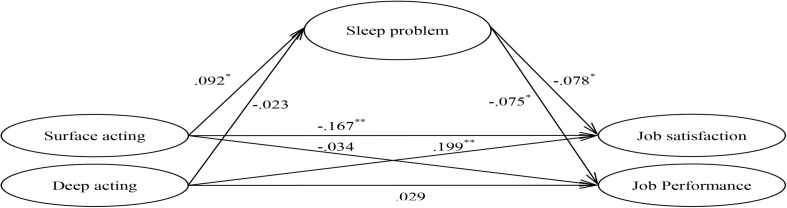
Results of structural model 3 analyses with standardized path coefficients. Age, gender, marital status, education, professional type, position, and Tenure were controlled. ^∗^*p* < 0.05, ^∗∗^*p* < 0.01.

Our results support that surface acting is significantly and negatively correlated with job satisfaction (γ = −0.167, *p* < 0.001) and deep acting is significantly and positively correlated with job satisfaction (γ = 0.199, *p* < 0.001). However, both surface acting and deep acting do not have a statistically significant correlation with job performance (Model 3, in Table 2 and see Figure 2).

In the context of the mediation hypotheses, the results indicate that surface acting is significantly and positively correlated with sleep problems (γ = 0.092, *p* = 0.025). In addition, sleep problems are significantly and negatively correlated with job satisfaction (β = −0.078, *p* = 0.032) and job performance (β = −0.075, *p* = 0.01) (Figure 2). As previously described, surface acting has a direct and negative effect on job satisfaction. This study also establishes the indirect effects of sleep problems on the surface acting–job satisfaction relationship (estimate = −0.007, *p* = 0.032, 95% CI [−0.021, −0.001]). Therefore, sleep problems only partially mediate the relationship between surface acting and job satisfaction (H_1a_). Our results reveal no direct effect of surface acting on job performance, but the surface acting–job performance relationship through sleep problems is significant (estimate = -0.007, *p* = 0.027, 95% CI [−0.019, −0.001], as shown in Table 3). Thus, we conclude that a complete mediating effect exists between surface acting and job performance (H_1__*a*_). Table 3 summarizes the total, direct, and indirect effects of surface acting on job satisfaction and job performance. As shown in Table 2 and Figure 2, Model 3 is a good fit to the data [χ^2^ (d.f. = 91) = 216.421, GFI = 0.976, CFI = 0.983, TLI = 0.964, RMSEA = 0.040, and SRMR = 0.025]. We find no mediating effect by sleep problems on the relationship between deep acting and job satisfaction and performance (H_1b_).

**TABLE 3:** Total, direct, and indirect effects from emotional labor to job satisfaction and job performance.

Paths	Estimate	SE	*p* value	95% CI
				**[upper bounds, lower bounds]**
**Effects from EL to JS**				
Direct: SA → JS	−0.167	0.045	0.003	[−0.251, −0.077]
Indirect: SA → SP → JS	−0.007	0.005	0.032	[−0.021, −0.001]
Total	−0.174	0.045	0.002	[−0.259, −0.087]
Effects from EL to JP				
Direct: SA → JP	−0.034	0.042	0.414	[−0.118, −0.051]
Indirect: SA → SP → JP	−0.007	0.005	0.027	[−0.019, −0.001]
Total	−0.040	0.042	0.321	[−0.126, −0.042]

*Standardized estimates and confidence intervals are presented with the bias-corrected bootstrapping procedures based on 5,000 samples. SE, standard errors; CI, confidence intervals; EL, emotional labor; JS, jab satisfaction; SA, surface acting; SP, sleep problem; JP, job performance.*

### Moderating Effects

To test the moderation hypotheses (H_2_–H_6_), we establish five latent interaction models (models 4–8 in Table 2). Each model separately examines the moderation of each personality trait in the relationship between emotional labor and sleep problems. The results suggest that extraversion has a significant moderating effect only on the relationship between deep acting and sleep problems (γ = 0.321, *p* = 0.029) for the product terms of extraversion and deep acting (figure not shown). Agreeableness significantly moderates only the relationship between surface acting and sleep problems (γ = −0.521, *p* = 0.046) for the product terms of agreeableness and surface acting (figure not shown). Conscientiousness does not play a significant moderating role between emotional labor and sleep problems (figure not shown). Neuroticism significantly moderates only the relationship between surface acting and sleep problems (γ = 0.201, *p* = 0.034) for the product terms of neuroticism and surface acting (figure not shown). Model 8 examines the moderating effect of openness and finds no significant moderating role between emotional labor and sleep problems (figure not shown). Table 2 reports the following results that indicate good fit for models 4–8: GFI ≥ 0.956, CFI ≥ 0.969, TLI ≥ 0.951, RMSEA ≤ 0.038, and SRMR ≤ 0.037. In sum, agreeableness and neuroticism moderate the relationship between surface acting and sleep problems, whereas extraversion moderates that between deep acting and sleep problems.

**FIGURE 3:**
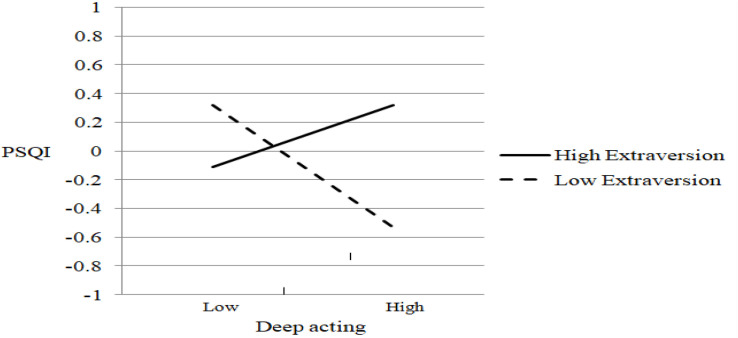
The moderating effect of extraversion on deep acting-sleep problems relationship.

**FIGURE 4:**
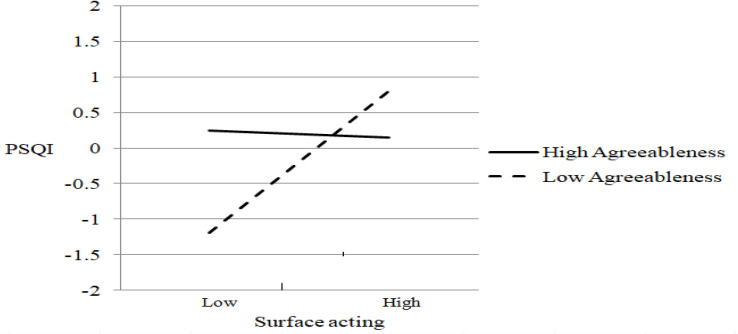
The moderating effect of agreeableness on surface acting-sleep problems relationship.

**FIGURE 5:**
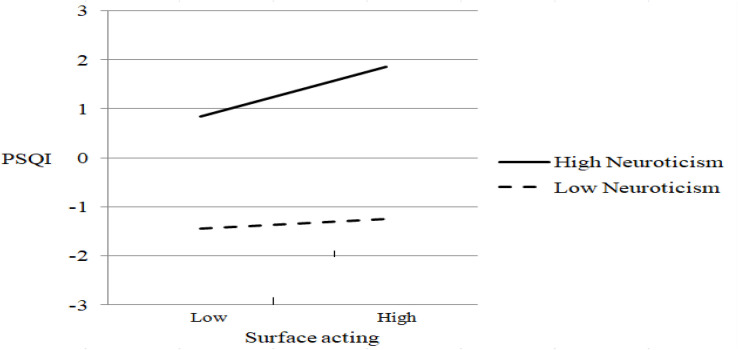
The moderating effect of neuroticism on surface acting-sleep problems relationship.

Figures 3–5 display the patterns of the moderating effects. The negative relationship between deep acting and sleep problems is stronger for low extraversion (simple slope test: β = −0.425, *t* (860) = −11.21, *p* < 0.001; Figure 3). The positive relationship between surface acting and sleep problems is stronger for low agreeableness (simple slope test: β = 0.997, *t* (860) = 3.87, *p* < 0.001; Figure 4) and high neuroticism (simple slope test: β = 0.507, *t* (860) = 2.72, *p* = 0.007; Figure 5). Conscientiousness and openness do not have any moderating effect in this study.

## Discussion

Our findings extend the emerging body of literature on emotional labor in several ways. First, we propose a mechanism that explains how job satisfaction and performance are influenced by emotional labor through sleep problems, which is prevalent in the working population. Our results indicate that only surface acting influences sleep problems, through which it further impacts job satisfaction and performance. This is consistent with meta analytic studies from Hülsheger and Shewe (2011) and Kammeyer-Mueller et al. (2013), surface acting is generally negatively related to impaired well-being, job attitude, and job performance, whereas deep acting has weak or no relations with well-being. Requiring employees to display emotions at work that are far from their genuine emotions may exhaust employees’ mental resources, stimulate negative emotions, and influence sleep quality. Since the psychological environment influences biological outcomes, such unpleasant emotional experiences during work are reported to have an influence on sleep (Greenberg, 2006). Supervisors who fail to recognize the influence of surface action on sleep are likely to be less prepared to tackle organizational problems (e.g., injuries, unethical behavior, and job performance) that plague sleep-deprived emotional laborers (Wagner et al., 2014).

Second, we found that the effects of surface acting on sleep problems are more pronounced among people with high (as opposed to low) neuroticism and low (as opposed to high) agreeableness for the following reasons: (a) surface acting involves suppressing, amplifying, or faking expressions (Hochschild, 1983; Grandey, 2003) which increases the probability of sleep problems, (b) individuals with high neuroticism are more likely to report fewer health-promoting behaviors (Booth-Kewley and Vickers, 1994), higher insomnia (Williams and Moroz, 2009), and more emotions (Watson and Clark, 1994), and (c) people with low agreeableness dislike being told what to do as this will pressure them into being inauthentic with their emotions, thereby developing sleep problems as a result (Hintsanen et al., 2014). Further, individuals with high neuroticism or low agreeableness develop more sleep problems when performing surface acting, whereas those with low neuroticism or high agreeableness remain relatively unaffected. Overall, the interactions between surface acting and high neuroticism or low agreeableness aggravate the adverse effects of surface acting on sleep problems.

Although some studies have shown higher extraversion to be related to better sleep quality (i.e., Gray and Watson, 2002; Williams and Moroz, 2009; Hintsanen et al., 2014; Allen et al., 2016), we did not find evidence in support of this relationship. In contrast to our hypothesis, our results suggest that extraversion acts as a moderator on the negative relationship between deep acting and sleep problems only among individuals with low extraversion. One possible explanation for such effect may be that people low on extraversion are better at conciliating gaps between the authentic and the required [self], or in this case “emotions,” as they may hold more experience doing so in their day-to-day lives to maintain a balance between socializing with the outer world and recharging via focusing on their internal thoughts and authentic feelings. Moreover, taking from sleep theory, we suspect that the effect of high extraversion on deep acting and sleep problems may be mitigated by the greater susceptibility of extraverts to experience emotional injury during social contexts, thereby creating stress and subsequent sleep problems after having engaged in social activities (Rupp et al., 2010). Overall, the effect of extraversion on sleep quality remains inconclusive, as other studies have also been unsuccessful in finding evidence to support such effect (Duggan et al., 2014; Kim et al., 2015; Huang et al., 2016; Cellini et al., 2017). Future studies are encouraged to continue exploring the moderating role of extraversion between deep acting and sleep problem.

Understanding the associations between personality traits and sleep can be useful in targeting those at an increased risk of sleep problems. Further, the findings can be particularly meaningful when recruiting personnel for jobs in which the lack of vigilance may have serious consequences. Individuals should consider their personality when selecting jobs to foster the person-job fit and person-organization fit (Holland, 1985). This may improve emotional labor and its impact on job satisfaction and performance. In sum, this study demonstrates that three out of five personality traits result in interactive effects by emotional labor on sleep. Examining personality traits and emotional labor contributes to the knowledge base necessary to map phenotypes for individual variations in sleep.

### Implications

Our results have several implications for both individuals and organizations. Emotional labor, a work demand, had a substantial influence on sleep problems, job satisfaction, and job performance. Therefore, employees could benefit from deep acting training that entails learning techniques of emotion regulation (Totterdell and Holman, 2003). Through such a training program, employees would be able to regulate their inner feelings to appear authentic to patients and clients more easily. Deep acting reduces emotional dissonance by aligning displayed emotions with felt emotions (Grandey et al., 2005), which, in turn, decreases sleep problems and increases job satisfaction and performance.

The belief that “the customer is always right,” that exists in many industries most likely results in higher levels of stress for employees. Workers are often asked to present a friendly demeanor and smiling face no matter how unsympathetic or unreasonable are their clients (Kammeyer-Mueller et al., 2013). In health care, the information asymmetry that exists between providers and patients often causes disputes. Asking health care professionals to show emotional labor will suppress their genuine emotions and will lead to detrimental consequences for their health. Therefore, training to show empathy help identify emotions in others, feel emotions, and comment appropriately on emotions. It may reduce misunderstanding between health care professionals and patients, and thus deviate the employee away from a situation that would require emotional labor.

We examined the moderating role of personality between emotional labor and sleep problems and suggest that understanding different personality traits and factors related to sleep problems may improve the individualized treatment of sleep problems. In addition, people should take these factors into consideration when selecting jobs and in fostering the person-job fit and person-organization fit. This may improve emotional labor and its impact on job satisfaction and job performance (Benoliel and Somech, 2014).

### Limitations

This study has several limitations and results should be interpreted with caution. First, sleep problems are measured using a complicated index, PSQI; however, this is not an objective scale. While it is advantageous to collect objective measures (e.g., episodes of waking during the night and the amount of rapid eye movement during sleep), such measurements can be obtained only in clinical settings. Given that we sought to examine the effects of sleep problems in a working sample of healthcare professionals, the collection of such objective measures was not feasible. Studies have shown that subjective measures of sleep problems are highly correlated with more objective measures such as polysomnographic reports (Tepas and Mahan, 1989). However, we suggest using an objective measure to substitute self-reporting measures in a future study.

Second, we employed a gradually common procedure – the parceling technique – to form multiple products of interaction for latent variables, however, it is to be noted that results may vary when including different interaction terms and therefore caution should be taken when interpreting these findings. Furthermore, future studies are encouraged to explore the effects of other interaction terms.

We adopted surface acting and deep acting, two common strategies, to regulate emotion requirements based on Grandey (2003) and Brotheridge and Lee (2002). However, other scholars such as Diefendorff et al. (2005) and Ashforth and Humphrey (1993) have proposed a third construct – in which employees can spontaneously or naturally experience, feel and display their true emotions. This display of naturally felt emotions is highly suggested for future research.

Additionally, the healthcare work environment influences the emotional requirements of the job; therefore, treating employees with shared social contexts as independent data points may not be appropriate (Grandey and Gabriel, 2015). Future studies may consider using an experience sampling method to capture the momentary changes in emotional regulation or investigating internal psychological processes of the individuals in the healthcare sector (Napa Scollon et al., 2009). Finally, stressors, such as patient incivility and family factors in making healthcare decisions, are highly prevalent in work settings. Future studies should control for these extraneous factors, given their potential influence on the relationship. It is also helpful to include measures for other emotional variables that are conceptually linked with sleep problems such as anxiety, depression, and anger (Arroll et al., 2012; Wagner et al., 2014).

## Conclusion

This study is an extension of the efforts to explain the mechanisms of emotional labor, job satisfaction, and job performance, a line of research that is becoming increasingly crucial given the high costs related to sleep problems and work-related outcomes in healthcare. It provides a distinct boundary condition, a mediating model, which incorporates sleep problems during work as a critical factor connecting emotional labor with job satisfaction and performance. The results suggest that being sleep deprived at work may negatively affect job performance and satisfaction. In particular, certain traits such as high neuroticism and low agreeableness aggravate the effects of surface acting on sleep problems, whereas low extraversion facilitates the relationship between deep acting and low sleep problems.

## Data Availability Statement

The raw data supporting the conclusions of this article will be made available by the authors, without undue reservation.

## Ethics Statement

The studies involving human participants were reviewed and approved by the Institutional Review Board of the Chi-Mei Medical Center (IRB No. 10109-001). The patients/participants provided their written informed consent to participate in this study.

## Author Contributions

S-CY: conceptualization, data curation, study design, methodology, and manuscript draft. S-HC: conceptualization, data curation, and manuscript draft. K-SY: data collection and formal analysis. WC: data collection and project administration. TW: review and editing. All authors contributed to the article and approved the submitted version.

## Conflict of Interest

The authors declare that the research was conducted in the absence of any commercial or financial relationships that could be construed as a potential conflict of interest.
